# PIN-G – A novel reporter for imaging and defining the effects of trafficking signals in membrane proteins

**DOI:** 10.1186/1472-6750-6-15

**Published:** 2006-03-08

**Authors:** Lynn McKeown, Philip Robinson, Sam M Greenwood, Weiwen Hu, Owen T Jones

**Affiliations:** 1 Faculty of Life Sciences, University of Manchester. 1.136 Stopford Building, Oxford Road, Manchester, M13 9PT, UK

## Abstract

**Background:**

The identification of protein trafficking signals, and their interacting mechanisms, is a fundamental objective of modern biology. Unfortunately, the analysis of trafficking signals is complicated by their topography, hierarchical nature and regulation. Powerful strategies to test candidate motifs include their ability to direct simpler reporter proteins, to which they are fused, to the appropriate cellular compartment. However, present reporters are limited by their endogenous expression, paucity of cloning sites, and difficult detection in live cells.

**Results:**

Consequently, we have engineered a mammalian expression vector encoding a novel trafficking reporter – pIN-G – consisting of a simple, type I integral protein bearing permissive intra/extracellular cloning sites, green fluorescent protein (GFP), cMyc and HA epitope tags. Fluorescence imaging, flow cytometry and biochemical assays of transfected HEK293 cells, confirm the size, topology and surface expression of PIN-G. Moreover, a pIN-G fusion construct, containing a Trans-Golgi Network (TGN) targeting determinant, internalises rapidly from the cell surface and localises to the TGN. Additionally, another PIN-G fusion protein and its mutants reveal trafficking determinants in the cytoplasmic carboxy terminus of Kv1.4 voltage-gated potassium channels.

**Conclusion:**

Together, these data indicate that pIN-G is a versatile, powerful, new reporter for analysing signals controlling membrane protein trafficking, surface expression and dynamics.

## Background

The identification of sequence motifs that direct proteins to appropriate intracellular and plasma membrane locales represents a fundamental objective of cell biology [1]. Once defined, such signals permit the identification of mechanisms underlying protein trafficking [2] and the targeted delivery of therapeutic agents to those subcellular domains where they are most effective [3]. Unfortunately, the identification of trafficking signals is not trivial as they may consist of non-contiguous amino acids, whose juxta-position to form a binding site may only be apparent in the fully folded protein [4]. Even in cases where trafficking signals are comprised of contiguous amino acids, they may be hard to identify since they lie within discrete subunits of complex oligomeric proteins [5]. Moreover, trafficking signals may overlap [6], act hierarchically [7], or be capable of being switched from inactive to active states through second messenger regulation [8].

**Figure 1:**
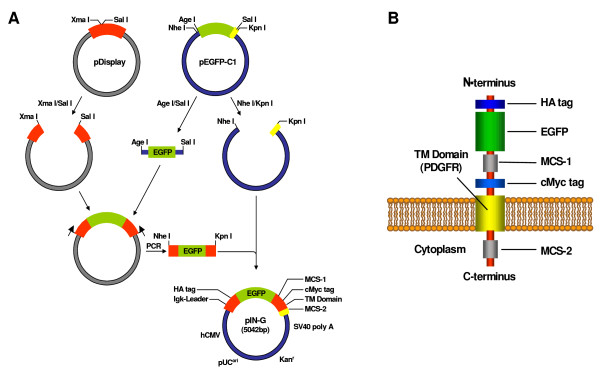
Cloning scheme for the preparation of the pIN-G construct and the transmembrane topology of the pIN-G reporter protein. **1A**. A fragment encoding EGFP, obtained by AgeI/SalI digestion of pEGFP-C1, was ligated into an XmaI/SalI digested pDisplay backbone and the entire protein coding region PCR-amplified and ligated into a host backbone generated through NheI/KpnI digestion of pEGFP-C1 to retain the 3' multiple cloning site but exclude the EGFP coding region. The final pIN-G construct, with the appropriate reading frame throughout, was generated via a subsequent round of PCR. See Methods for further details. **1B**. Following expression and cleavage of the Igk- chain leader sequence, the pIN-G protein is proposed to adopt a transmembrane orientation where HA and cMyc epitope tags and GFP have an extracellular disposition at the cell surface (or intra-lumenal in organelles or vesicular trafficking intermediates).

A well-established strategy to identify trafficking signals has been to infer their necessity and sufficiency from their ability to direct simpler reporter proteins to which they are fused, to the appropriate cellular compartment [9-11]. Unfortunately, available reporter proteins have numerous limitations including their endogenous expression in diverse cells and tissues, lack of convenient cloning sites and potential interactions with intra- or extracellular proteins that may affect their subcellular distributions [12]. Even more significant is the limited capacity for detection of presently available reporters, not least because of their reliance upon the limited range of antibodies that are commercially available and the indirect nature of the detection methodology. To circumvent such problems, we have engineered an alternative protein trafficking reporter system (Fig. 1) consisting of a simple artificial type I integral protein bearing permissive intra- and extracellular multiple cloning sites, a green fluorescent protein moiety (GFP) that permits direct localisation by fluorescence imaging in live cells and epitope tags for biochemical detection of protein turnover and surface expression.

## Results

### Vector construction

To generate a suitable trafficking reporter (Fig. 1), a region encoding EGFP and unique extracellular restriction sites from pEGFP-C1 (Clontech) was first inserted into the cell surface expression vector, pDisplay, (Invitrogen) to generate an intermediate EGFP-pDisplay construct (Fig. 1A). The entire protein coding region of EGFP-pDisplay was then PCR amplified and cloned into a pEGFP backbone lacking the EGFP insert, thereby introducing a second, distinct, intracellular multiple cloning site (MCS-2). The primary homologue, described henceforth as pIN-G, is a 5042bp pUC-based vector (see Table 1 for details) containing all the desired protein coding elements in the correct reading frame (Fig. 1B)(deposited in Genbank as Accession number AY841887).

### Properties of pIN-G

**Figure 2:**
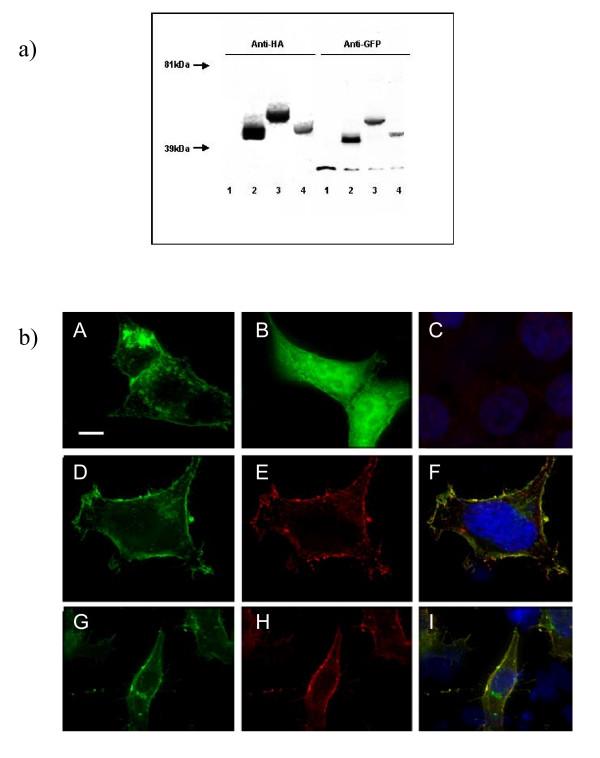
**a) **pIN-G and fusion protein constructs yield proteins of the anticipated sizes following expression in HEK293 cells. Detection of pIN-G protein (lane 2) through immunoblotting (see Methods) with antibodies against HA and GFP reveals a protein of 41 kDA. Lanes 3 and 4 correspond to pIN-G fusion proteins bearing the carboxy terminus of the Kv1.4 potassium channel (pIN-JHE811) or TGN38 trans-Golgi Network targeting motif (pIN-TGN38), respectively. Lane 1 corresponds to mock-transfected cells. **b) **Total and surface expression of pIN-G in transfected HEK293 cells determined through fluorescence imaging. HEK293 cells transiently transfected with pIN-G (A) or EGFP (B) were fixed and viewed with a 100× objective using a DeltaVision workstation (Methods) (Panel C denotes background fluorescence of mock-transfected cells upon omission of primary antibody). Panels D-I, denote total (D and G) versus surface (E and H) expression of pIN-G determined through treatment of non-permeabilised pIN-G-transfected cells with antibodies to EGFP (E), and HA (H), followed by fixation and labeling with the appropriate Cy3-conjugated secondary antibody. Panels F and I indicate merged images. Green: pIN-G fluorescence; blue: DAPI (nuclei); red Cy3 2° antibody. Areas of red/green overlap are shown in yellow. Scale bars correspond to 15 μM.

Transfection of pIN-G into HEK293 cells, and subsequent immunoblotting with antibodies against EGFP and HA disclosed a novel 41.3 kDa band, which was absent from mock-transfected cells (Fig. 2a). Constructs encoding pIN-G fused to a TGN targeting motif and the carboxy terminus of the voltage-gated potassium channel Kv1.4 gave bands of (44 kDa) and (51 kDa), respectively.

**Figure 3:**
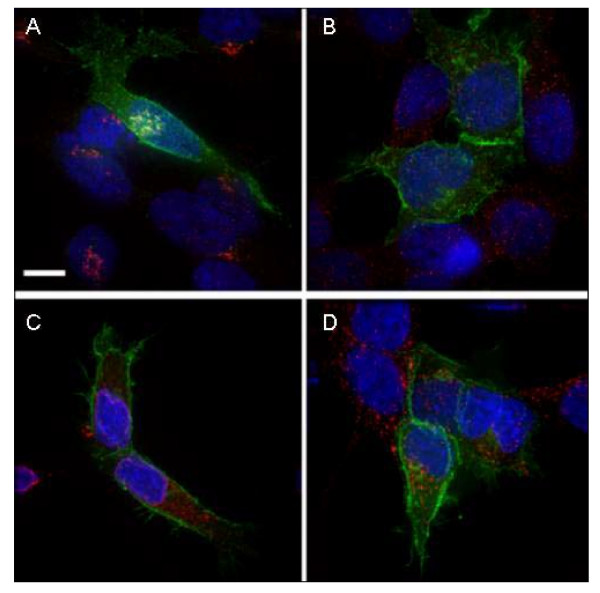
Intracellular distribution of PIN-G in membrane compartments. HEK293 cells transiently transfected with pIN-G, were fixed, permeabilised and treated with antibodies (See Methods) to the following organelle markers: A, GM130 (Golgi apparatus); B, EEA1 (Early endosomes); C, Calnexin (Endoplasmic Reticulum) and D, LAMP-1 (Lysosomes) followed by appropriate Cy-3 conjugated secondary antibody. Green denotes pIN-G fluorescence while red corresponds to the organelle marker. Areas of red/green overlap are shown in yellow. Blue indicates DAPI-stained nuclei. Scale Bar: 15 μM.

Fluorescence images of pIN-G-transfected HEK293 cells (Fig. 2b panel A) revealed strong green heterogeneous fluorescence within intracellular organellar structures and at the plasma membrane. In contrast, EGFP alone (Fig. 2b panel B) was expressed homogeneously throughout the cell. Thus, the presence of an Igκ leader sequence and the PDGF receptor transmembrane region, but no added trafficking motifs, appeared to direct pIN-G via the secretory pathway to the cell surface. To confirm this, first, the surface expression of pIN-G was tested by immunostaining non-permeabilized fixed cells with anti-EGFP (Fig. 2b panels D-F) and anti-HA (Fig. 2b panels G-I), and subsequent detection with Cy3-labeled secondary antibodies. In each case, Cy-3 (red) fluorescence overlapped strongly with pIN-G fluorescence at the cell margin, and was absent upon omission of primary antibody to mock-transfected cells (Fig. 2b panel C). Next, the intracellular disposition of pIN-G was resolved through immunocytochemistry with established antibody markers (in parentheses) to membranous organelles (Fig. 3A–D) including (A) the Golgi apparatus (GM130) [16], (B) early endosomes (EAA-1) [17], (C) the endoplasmic reticulum (ER; calnexin) [18] and (D) the lysosomes (LAMP-1)[19]. Throughout, we found no evidence for cytotoxicity or inclusion body formation.

### pIN-G is a faithful trafficking reporter

**Figure 4:**
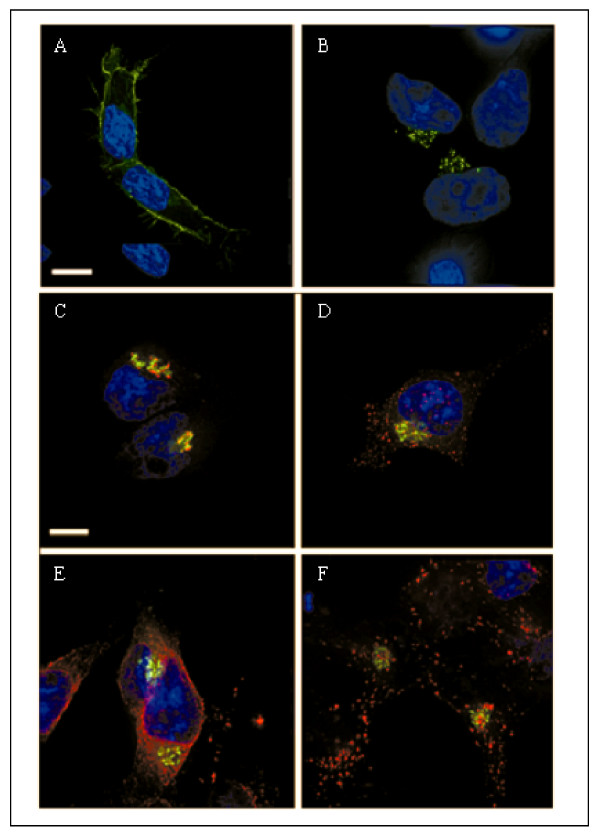
Re-specification of the distribution of pIN-G by an autonomous targeting signal. HEK293 cells transiently transfected with pIN-G or pIN-TGN38 – a fusion construct corresponding to pIN-G bearing the autonomous targeting signal of the Trans-Golgi Network resident protein TGN-38 – were fixed, stained and imaged on a Delta-Vision workstation. Panel A, pIN-G; Panel B, pIN-TGN. HEK293 cells transiently transfected with pIN-TGN38, were fixed, permeabilised and treated with antibodies (See Methods) to the following organelle markers: C, GM130 (Golgi apparatus); D, EEA1 (early endosomes); E, Calnexin (endoplasmic reticulum) and F, lamp-1 (Lysosomes) followed by the appropriate Cy-3 conjugated secondary antibody. Green denotes pIN fluorescence while red corresponds to the organelle marker. Areas of red/green overlap are shown in yellow. Blue indicates DAPI-stained nuclei. Scale Bar: 15 μM.

Next, we sought to establish the potential of pIN-G as a reporter of protein trafficking by exploiting the observation that a contiguous carboxy terminal motif within TGN-38 is both necessary and sufficient to localise this protein to the Trans-Golgi Network (TGN)[20]. Thus, cDNA encoding the TGN-38 signal with a corresponding stop codon, was cloned into the putative intracellular multiple cloning site of pIN-G (MCS-2) to yield a construct, designated as pIN-TGN. Fluorescence imaging of HEK293 cells transfected with pIN-TGN (Fig. 4), revealed a high concentration of fluorescence in vesicles and membranes distributed proximal to the nucleus in a characteristic 'signet ring' pattern (Fig. 4B) distinct from that seen with either pIN-G (Fig. 3A–D and Fig. 4A) or EGFP (Fig. 2b panel B). Further immunocytochemistry showed extensive co-localisation of pIN-TGN with the Golgi apparatus, but not markers of early endosomes, the ER or lysosomes (Fig 4C–F, respectively).

### Analysis of pIN-G surface expression levels

**Figure 5:**
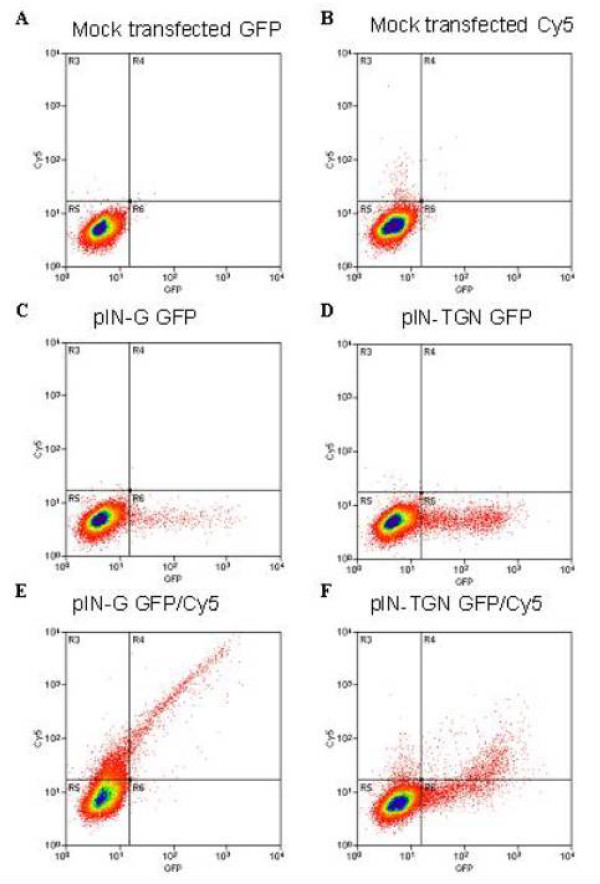
Differences in the surface levels of pIN-fusion proteins determined by flow cytometry. HEK293 cells transfected with no cDNA (mock, panel A and B), pIN-G (panel C and E) or pIN-TGN (panel D and F) were subjected to FACS analysis either with (panel B, E and F) or without (panel A, C and D) anti-HA antibody followed by detection with Cy5-conjugated goat anti-mouse IgG_1 _(Methods). Calculated mean fluorescent intensity (MFI) values determined the background fluorescence (panel A) and non-specific antibody binding (panel B) of mock transfected cells. Panel C and D demonstrate the specific GFP fluorescence of pIN-G or pIN-TGN transfected cells respectively (quadrant R5), whilst surface labeling of transfected cells, with anti-HA and Cy5 primary and secondary antibodies reveal a cell population displaying both red and green fluorescence (panels E and F, quadrant R4).

To confirm the cell surface disposition of pIN-G and pIN-fusion constructs, and its facile resolution by cell sorting assays, FACS analysis was used. In mock-transfected cells (Fig. 5A), or those treated with Cy5 secondary antibodies alone (Fig. 5B), most cells showed low fluorescence (Fig. 4, quadrant R3 (red) and R6 (green)). However, upon transfection with pIN-G or pIN-TGN, a new population of cells was revealed that showed strong fluorescence in the green channel, (Fig. 5C, 5D, quadrant R6). After surface labelingof pIN-G and pIN-TGN transfected cells, with anti-HA and Cy5 primary and secondary antibodies, a novel cell population displaying both red and green fluorescence (Fig. 5E and 5F, quadrant R4) emerged, corresponding to those expressing construct at the cell surface. By defining F as the fraction of transfected cells in the R4 quadrant (i.e. F = R4/(R4+R6)), pIN-G surface expression was much greater than that of pIN-TGN (F = 0.99 vs. 0.44, respectively).

### A pIN-G fusion protein identifies internalization determinants in the Kv1.4 carboxy terminus

**Figure 6:**
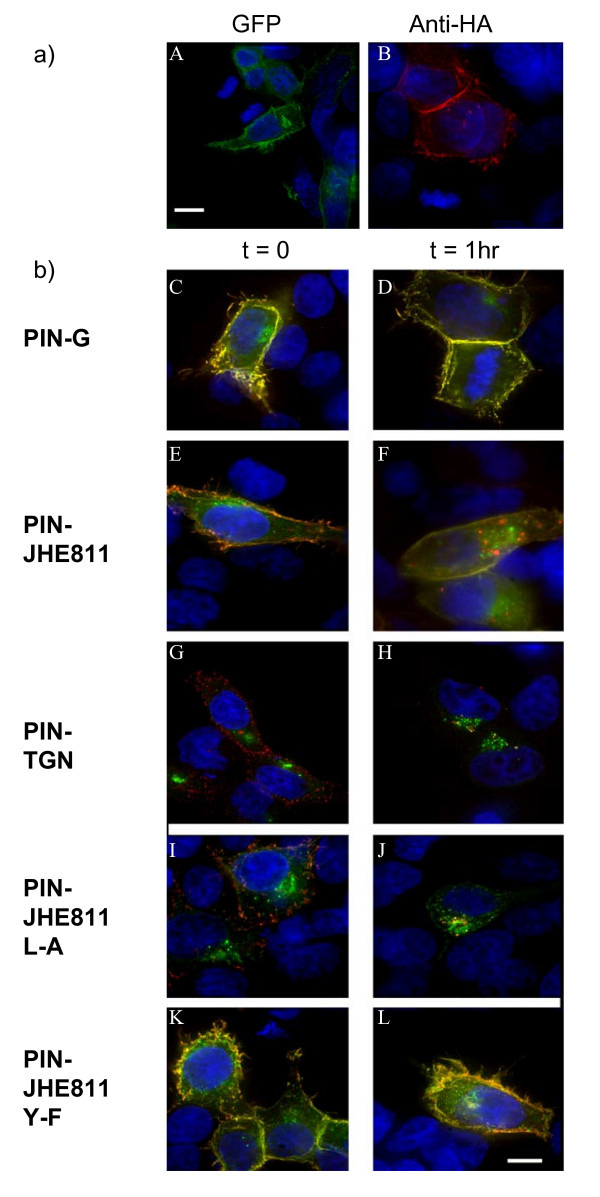
**a) **Total versus surface expression of pIN-JHE811 – a fusion construct corresponding to pIN-G bearing the cytoplasmic carboxy terminus of Kv1.4. HEK293 cells transiently transfected with pIN-JHE811 were fixed, stained and imaged on a Delta-Vision workstation. Panel A demonstrates total cellular pIN-JHE811 fluorescence (green) whilst panel B depicts surface staining of pIN-JHE811 with anti-HA antibodies (red: as described in Methods). Blue indicates DAPI-stained nuclei. **b) **Site-directed mutagenesis of pIN-JHE811 reveals internalization signals in the Kv1.4 carboxy terminus. HEK293 cells transiently transfected with pIN-G (panels C and D), pIN-JHE811 (panels E and F), pIN-TGN (panels G and H), pIN-JHE811 L-A (panels I and J) and pIN-JHE811 Y-F (panels K and L) and were labeled with anti-HA antibodies and either fixed directly (panels C, E, G, I and K) or incubated for 1 hour at 37°C to allow antibody internalization (panels D, F, H, J and L). Internalized antibody was then detected using Cy3-conjugated 2° goat anti-mouse antibody. Green denotes PIN-protein fluorescence while red corresponds to the anti-HA antibody. Areas of red/green overlap are shown in yellow. Blue indicates DAPI-stained nuclei. Scale Bar: 15 μM.

Available evidence suggests that Kv1 family potassium channels contain one or more carboxy terminal internalization determinants [15,21]. To identify such determinants, a construct was generated encoding pIN-G to which the entire carboxy terminus of rat Kv1.4 (Genbank accession no. NM 012971) had been fused (pIN-JHE811). In transfected HEK293 cells, pIN-JHE811 fluorescence was localized to intracellular membrane vesicles and the cell surface (Fig. 6a, panel A). Surface expression of pIN-JHE811 was confirmed through immunocytochemistry of non-permeabilised cells treated at 4°C with antibodies raised against the HA epitope tag (see Fig. 6a, panel B). However, in contrast to pIN-G (Fig. 6b, panel C), pIN-JHE811 surface labeling appeared less intense as indicated by a decrease in green:red (yellow) surface staining (Fig. 6b, panel E). Since, pIN-JHE811 contains putative internalization motifs, we questioned whether such diminished surface labeling at steady-state compared to pIN-G, might reflect partial redistribution of pIN-JHE811 into the endocytic pathway. To test this notion, we determined the fate of surface-labeled pIN-JHE811 compared to pIN-G following a pulse chase-protocol with anti-HA antibody (Fig. 6b panels C-F). After 1 h of internalization prior to labeling with Cy3-conjugated secondary antibodies (see Methods), cells expressing pIN-JHE811 showed diminished surface labeling compared to cells fixed and labeled immediately after anti-HA application i.e. at time 0 h (Figs. 6b panels E, F). In contrast, a similar high level of surface labeling was seen at both t = 0 h and 1 h for cells expressing pIN-G (Figs. 6b panels C, D). Since TGN-38 is routed via the surface to the TGN [13,14], we also tested internalization of anti-HA in cells expressing pIN-TGN. As shown in Figs. 6b panels G and H, surface labeling of pIN-TGN, while weak at t = 0 h, was negligible after 1 h.

Inspection of the C-terminal sequence of Kv1.4 reveals the presence of several candidate internalization motifs (notably di-leucine Leu-594/Leu-595 and Tyr-612). In anti-HA pulse-chase experiments, a di-leucine mutant, Leu594Ala (pIN-JHE811 L-A), surprisingly, showed enhanced intracellular and diminished cell surface Cy3-labeling compared to pIN-JHE811 (Fig. 6b panels I and J). In contrast, a Tyr612-Phe (pIN-JHE811 Y-F) mutant showed enhanced surface and reduced intracellular labeling (Fig. 6b panels K and L). FACS analysis of cells expressing pIN-JHE811 mutants at t = 0 and 1 hr yielded internalization half-lives commensurate with the imaging data of 160mins, 40 mins and infinity for pIN-JHE811; L-A and Y-F, respectively. Thus, like the homologous residue (position 457) in Kv1.2, tyrosine 612 serves as a critical internalization signal [21].

## Discussion

In this paper, we describe a novel construct – pIN-G – for the facile detection of trafficking determinants within membrane proteins. Expression of pIN-G yields an artificial, GFP-tagged, protein – PIN-G – whose characteristics and topology conform to the type I protein depicted in Figure 1B. Thus, PIN-G is recognised by surface-applied antibodies corresponding to each extracellular moiety and is also re-distributed by sequences appended to the putative intracellular domain. The presence of PIN-G at the cell surface (and throughout membranes of the secretory pathway) also suggests that it does not contain any intrinsic targeting or trafficking determinants. However, the addition of sequences containing known or putative targeting determinants re-specifies the trafficking fate of the expressed fusion construct indicating that pIN-G is a faithful reporter for visualising and analysing trafficking mechanisms. Equally salient is the utility of the pIN-G system in identifying and dissecting motifs, such as the previously undocumented Tyr612 in Kv1.4, that control the dynamics and expression of cell surface proteins, especially when, like Kv1.4, they have no specific radioactive ligands or where biotinylation strategies may not be straightforward.

In contrast to existing reporters [9-11], the pIN vector is a considerably more versatile tool, not least because of the presence of intra- and extracellular multiple cloning sites, epitope and GFP tags and lack of endogenous expression. Moreover, unlike with present reporters, any aberrant expression of pIN-G or its fusion constructs (such as inclusion body formation), while absent in the present study, should be readily visualised. Such versatility could be extended even further to include the dynamic visualisation of interacting proteins in live cells [22], and the development of combinatorial library approaches for identifying intracellular or extracellular signalling mechanisms affecting trafficking [23]. Other potential applications of pIN technology include the generation of constructs with alternate fluorophores to GFP such as mRFP [24], thereby, permitting the development of biosensors or energy transfer assays between pIN-fusion constructs. Finally, the reasonably small size and simplicity of the pIN construct, especially when compared to most integral proteins mediating signal transduction, should facilitate the analysis of trafficking in cells such as neurons which are hard to transfect [25] and also the development of viral gene delivery methodologies where packaging size is critical [26].

## Conclusion

Here we have described the construction and faithful expression of a novel trafficking reporter protein, specifically designed for the detection and characterization of autonomous trafficking motifs. By incorporating epitope and GFP tags into a simple type I transmembrane protein, our construct permits the direct visualization and biochemical dissection of protein signals and signalling domains transplanted from much more complex parent membrane proteins. The potential uses of the pIN-G reporter are, however, not limited to trafficking studies and could serve as a platform for the development of novel biosensors and the high-throughput screening of protein-protein interactions.

## Methods

### Materials

A clone encoding GFP- tagged TGN-38 (pΔMep4-TGN-38-GFP) was a gift from Dr. G. Banting, (Univ. Bristol, UK). Monoclonal antibodies GM130, anti-EEA-1 and anti-calnexin were purchased from BD Biosciences Pharmingen, Oxford, U.K. and anti-HA.11 from Covance Research Products, Berkeley, U.S.A. The 9E10 anti-cMyc antibody was a gift from Prof. M. Humphries lab (Manchester, U.K.) and anti-TGN46 a gift from Dr. M. Lowe (Manchester, U.K.). The Lamp-1 antibody (clone H4A3) developed by August and Hildreth was obtained from the Developmental Studies Hybridoma Bank, Iowa, U.S.A. Polyclonal antibodies raised against GFP (A11122) were obtained from Molecular Probes, Invitrogen, Paisley, U.K. Immunoblotting reagents were from Amersham Pharmacia Biotech, Bucks, U.K. Oligonucleotides were synthesized by ACGT Inc., Toronto, ON, Canada or Sigma-Genosys, U.K. All other chemicals were of reagent grade or higher purity.

### pIN-G vector construction

Generation of the pIN-G construct (Fig. 1B) was achieved by first digesting the pEGFP-C1 vector (Clontech, Palo Alto, CA) with Age I (bp 601) and Sal I (bp 1370) to yield a 769 bp fragment, corresponding to the coding region of EGFP. The resulting fragment was then subcloned into the Xma I (bp 849) and Sal I (bp 869) sites of the pDisplay vector (Invitrogen, San Diego, CA) to give a 6074 bp intermediate EGFP-pDisplay construct. The entire protein coding region of EGFP-pDisplay (bp732–1803) was then subject to PCR amplification using the following forward and reverse oligonucleotide primers: 5'-ATC CGC TAG CGC TAC TAG TCG CCA CCA TGG AGA CAG ACA CAC TC-3' and 5'-AAA GAC CGG TGA ACG TGG CTT CTT CTG CCA AAG CAT-3' which also served to introduce Nhe I and Kpn I sites for subcloning. Separately, the entire EGFP-coding region was excised from pEGFP-C1 by digestion with Nhe I (bp592) and Kpn I (bp1380). The linearized pEGFP-C1 backbone was then used as a host into which the EGFP-pDisplay PCR fragment, generated above, was subcloned. The resulting 5037 bp pIN precursor construct (pIN-G') was then used to generate pIN construct derivatives. For example, pIN-G was constructed by generating two PCR fragments pIN-A (401 bp), containing SnaB I and Pvu I sites for subcloning, (forward primer: 5'-GGA CTT TCC TAC TTG GCA GTA C-3'; reverse primer: 5'-TTT TCG ATC GCA GCA TAA TCT GGA ACA TCA TAT GG-3') and pIN-B (996 bp), containing Pvu I and Kpn I sites for subcloning, (forward primer: 5'-AAA ACG ATC GAA CCC AGC CGG CCA GAT CT-3'; reverse primer: 5'-AAA GGG TAC CGA ACG TCG CTT CTT CTG CCA AAG CAT-3') from the pIN-G' template. Fragments pIN-A and pIN-B were then digested with Pvu I, ligated to give a 1383 bp fragment which was then cloned into the corresponding Snab I and Kpn I sites in pIN-G' to yield a correctly-framed pIN-G product (5042 bp) containing a novel Pvu I site.

### pIN-TGN-38 vector construction

The pIN-TGN-38 construct, was generated by PCR amplification of the sequence encoding the trans-Golgi-Network targeting signal (SEQYDRL: refs [13,14]) using pΔMep4-TGN-38-GFP as a template and the following forward and reverse oligonucleotide primers: 5'-AGA AGC CAC GTT CGC ACA ACA AAC G-3' and 5'-GAT CCC GGG CCC GCG TCA AAG CTT TAG G-3' that contained 15 bp homology to bases corresponding to the Kpn I site of pIN-G. The resulting PCR fragment was then cloned into the Kpn I site in the intracellular multiple cloning site (MCS-2; see Fig. 1B) of pIN-G by homologous recombination (BD-Dry Down In-Fusion PCR kit, Becton-Dickinson, UK).

### pIN-JHE811 vector construction

A pIN fusion construct, encoding pIN-G fused to the entire intracellular carboxy terminus of the Kv1.4 voltage-gated potassium channel, was generated by PCR using pEYFP-Kv1.4 [15] as a template and the following forward and reverse oligonucleotide primers: 5'-AAG TCT CTC TCG GGC TTC AGC -3' and 5'- AAG TCT CTC TCG GGC TTC AGC -3' that contained flanking sequences corresponding to the Kpn I site of pIN-G. The resulting PCR fragment was then cloned into the Kpn I site in MCS-2 and ligated using T4 ligase as described [15]. pIN-JHE811-L-A and pIN-JHE811-Y-F were created using the Stratagene QuikChange site-directed mutagenesis kit with pIN-JHE811 as a template and the following mutagenic oligonucleotide primers: 5'-CA TAC CTA CCT TCT AAT TTG CTC AAG AAA TTT CGG AGC TCT AC -3' and 5'-CTG GGG GAC AAG TCA GAG TTT CTA GAG ATG GAA GAA GGG-3'. The identity of all constructs was confirmed by restriction mapping and DNA sequencing (ACGT, Toronto, Canada, Geneblitz, Sunderland, UK).

### Tissue culture

HEK293 cells were subcultured every 3–4 days, incubated at 37°C in a humidified incubator and maintained with 5% CO_2_. Cells were detached from the flasks with phosphate-buffered saline without calcium and magnesium (PBS^-^), quenched in fresh medium, and re-seeded into T75 cm^2 ^flasks. Cells were cultured in Dulbecco's minimal essential medium (DMEM) containing 0.11 g/L sodium pyruvate and pyridoxine and supplemented with 10% FBS (v/v), 2 mM L-glutamine (w/v) and 1% penicillin and streptomycin.

### Transfection

All transfections were carried out using Fugene 6 (Roche, UK), according to the manufacturers' instructions. HEK293 cells were seeded at 1:40 from a confluent T75 into a 6-well plate and grown to 50% confluency overnight at 37°C. 2 μg cDNA was added to 150 μl DMEM and mixed in an eppendorf tube containing 6 μl of Fugene6 reagent and 150 μl DMEM. The cDNA and reagent solutions were combined to allow the formation of lipid complexes for 20 minutes at room temperature. Subsequent to the addition of fresh DMEM, to remove any antibiotics, the DNA-lipid complexes were added to the cells in a drop wise manner and incubated for 12 hours at 37°C. After this period cells were either transferred to a 10 cm dish for further propagation or re-seeded 1:4 onto collagen-coated coverslips in 12-well plates then incubated at 37°C overnight.

### Immunoblotting

HEK293 cells were lysed in RIPA buffer containing a cocktail of protease inhibitors (Roche, UK), and passed 8X through a 22-gauge needle to shear nucleic acids. The sheared lysate was then centrifuged at 16,000 gav for 15' and aliquots of the supernatant electrophoresed, and transferred to nitrocellulose and processed as described previously [15] using monoclonal antibodies to HA (1:1000)), or polyclonal antibodies to GFP (1:10,000). Immunoreactivity was detected by incubating each membrane with horseradish peroxidase-conjugated donkey anti-mouse or anti-rabbit (1:1000; Amersham, UK) followed by enhanced chemiluminescence (ECL; Amersham Biosciences) and film detection (Amersham Bio-Max).

### Immunostaining of transfected HEK293 cells

Image-based detection of PIN-fusion proteins was observed in HEK293 cells transiently transfected with pIN-G, pIN-TGN or pIN-JHE811, fixed in 4% paraformaldehyde for 20 minutes at room temperature, washed in PBS, quenched with 0.1 M glycine and mounted onto glass slides using ProLong Gold anti-fade (Molecular Probes). The surface expression of PIN proteins was determined by incubating non-permeabilised, fixed cells with anti-HA antibodies (1:1000) for 30 minutes at room temperature and detecting with Cy3-conjugated goat anti-mouse secondary antibody (1:200, Jackson Immunoresearch, Stratech Scientific, Cambs, UK). Intracellular staining of organelles was performed by fixing HEK293 cells in 4% paraformaldehyde (as above), permeabilising with 0.5% saponin for 5 minutes followed by incubation with antibodies raised against the appropriate markers at a concentration of 10 μg/ml in 0.01% saponin. Detection of specific staining was performed with Cy3-conjugated anti-mouse secondary at 1:200.

### Internalization assays

Internalization of the PIN fusion proteins was determined by pulse-labeling transfected HEK293 cells at 4°C with anti-HA antibodies (1:1000) for 30 minutes. The cells were then either fixed directly using 4% paraformaldehyde and permeabilised with 0.5% saponin, or incubated for 1 hr at 37°C in fresh medium prior to fixation and permeabilisation. For imaging, internalized anti-HA antibody was detected with Cy3-conjugated goat anti-mouse secondary (1:200) and nuclei were DAPI-stained (0.05 μg/ml) prior to mounting. For FACS analysis, to distinguish surface labeling transfected cells were not permeabilised and external anti-HA antibody was detected with Cy5-conjugated goat anti-mouse secondary (1:200)

### Fluorescent activated cell sorting (FACS)

For surface analysis, transfected cells were detached with PBS without calcium and magnesium (PBS^-^) and adjusted to a concentration of 5 × 10^7^/ml with DMEM containing 1% FBS (v/v). 100 μl aliquots of cells were incubated with primary antibody (anti-HA) at a concentration of 10 μg/ml diluted in Dulbecco's phosphate-buffered saline with calcium and magnesium (PBS^+^) for 30 minutes at 4°C. For pulse-chase assays, transfected cells were plated onto 10 cm cell-culture dishes and incubated overnight at 37°C. The dishes were placed on ice and the cells labeled with anti-HA at a concentration of 10 μg/ml diluted in PBS^+ ^for 30 minutes at 4°C. Cells were then detached and transferred to FACS tubes. After three washes with 300 μl PBS^+^, 1% FBS (v/v), all samples were incubated with Cy5-conjugated goat secondary anti-mouse IgG_1 _(1:200) diluted in 10% FBS and 90% PBS^+ ^(v/v) for 45 minutes at 4°C. Cells were fixed with 100 μl aliquots of 2% (w/v) formaldehyde, 100 μl PBS^-^, 300 μl PBS^+^. The dilutions of antibodies used were saturating as confirmed by titration. Cells (20,000 per sample) were analysed in a FACScaliber^® ^flow cytometer (Becton Dickinson, UK) and mean fluorescent intensity values taken. Internalization half-lives were determined assuming a first-order exponential.

### Delta Vision deconvolution microscopy

Mounted coverslips were visualized on an Olympus IX-70 inverted microscope using a ×100 UPLAN objective (NA 1.35) and images captured on a Photometrics CH350L CCD camera. The Delta Vision restoration microscope (Applied Precision Instruments, Seattle) utilized a Unix-based computer system equipped with SoftWoRx version 2.50, acquisition software. Epifluorescence was recorded using filter sets for DAPI/FITC/Texas red and cy3/cy5. Using the high precision nanochassis stage, widefield optical sections of 0.2 μm were acquired throughout the z plane of the cells. Z stacks generated were then deconvolved using a constrained iterative algorithm assigned by Delta Vision.

## Abbreviations

HEK293, human embryonic kidney 293; DMEM, Dulbecco's minimal essential medium; FBS, Fetal bovine serum; GFP, green fluorescent protein; PBS, phosphate-buffered saline; PMSF, phenylmethanesulfonyl fluoride; SDS-PAGE, sodium dodecyl sulfate polyacrylamide gel electrophoresis; HEPES, N-(2-hydroxyethyl) piperazine N'-(butanesulfonic acid); BSA, bovine serum albumin.

## Authors' contributions

LM carried out all imaging and FACs analysis, created the molecular pIN-JHE811 mutations and helped to draft the manuscript. PR made the pIN-TGN construct. SG characterized the cellular expression of pIN-G and its derivatives. WH performed molecular biology of pIN-G. OTJ conceived the idea, participated in its design and molecular biology and helped to draft the manuscript. All authors read and approved the final manuscript.

## Figures and Tables

**Table 1:** Features of the pIN-G reporter system. Features of the pIN-G Trafficking Reporter Plasmid. Nucleotide positions of the corresponding features of pIN-G are indicated in the right hand column. Unique restriction sites for the extracellular and intracellular Multiple Cloning Sites (MCS-1 and MCS-2, respectively) are italicised. Asterisks denote enzymes yielding blunt ends.

**Feature**	**Position (bp)**
Human CMV promoter	1–589
Enhancer region	59–465
AP1 binding site	400–407
CAAT box	521–525
TATA box	554–560
Transcription start site	583
Kozak translation initiation site	606–612
pIN-G start codon	613–615
Igκ-chain leader sequence	613–675
Haemagglutinin epitope tag	676–702
Unique Pvu I site	708
Enhanced Green Fluorescent Protein (EGFP)	742–1458
MCS-1 (BsrGI,AccIII,XhoI,Ecl136I*,SacI,HindIII,EcoRI,PstI,SalI)	1451–1504
c-Myc epitope tag	1504–1533
PDGFR transmembrane domain	1537–1685
MCS-2 (KpnI, SacII,ApaI, SmaI*,BamHI,XbaI,BclI)	1686–1718
pIN-G stop codon	1723–1725
SV40 early mRNA polyadenylation signals	1861–1866,1890–1895
mRNA ends	1899,1911
F1 single-strand DNA origin	1958–2413
Kan^r ^bacterial promoter	2475–2480,2498–2503
Kan^r ^transcription start point	2510
SV40 early promoter (tandem 72 bp)	2587–2730
SV40 origin of replication	2754–2889
Kanamycin/neomycin resistance gene	2938–3732
Herpes simplex thymidine kinase poly-A signal	3968–3973,3981–3986
pUC plasmid origin of replication	4317–4960
